# Cardiolipin, Mitochondria, and Neurological Disease

**DOI:** 10.1016/j.tem.2021.01.006

**Published:** 2021-04

**Authors:** Micol Falabella, Hilary J. Vernon, Michael G. Hanna, Steven M. Claypool, Robert D.S. Pitceathly

**Affiliations:** 1 Department of Neuromuscular Diseases, University College London Queen Square Institute of Neurology, London, UK; 2 Department of Genetic Medicine, Johns Hopkins University School of Medicine, Baltimore, MD, USA; 3 Department of Neuromuscular Diseases, University College London Queen Square Institute of Neurology and The National Hospital for Neurology and Neurosurgery, London, UK; 4 Department of Physiology, Johns Hopkins University School of Medicine, Baltimore, MD, USA

**Keywords:** cardiolipin, lipids, mitochondria, mitochondrial disease, nervous system, neurodegeneration

## Abstract

Over the past decade, it has become clear that lipid homeostasis is central to cellular metabolism. Lipids are particularly abundant in the central nervous system (CNS) where they modulate membrane fluidity, electric signal transduction, and synaptic stabilization. Abnormal lipid profiles reported in Alzheimer’s disease (AD), Parkinson’s disease (PD), amyotrophic lateral sclerosis (ALS), and traumatic brain injury (TBI), are further support for the importance of lipid metablism in the nervous system. Cardiolipin (CL), a mitochondria-exclusive phospholipid, has recently emerged as a focus of neurodegenerative disease research. Aberrant CL content, structure, and localization are linked to impaired neurogenesis and neuronal dysfunction, contributing to aging and the pathogenesis of several neurodegenerative diseases, such as AD and PD. Furthermore, the highly tissue-specific acyl chain composition of CL confers it significant potential as a biomarker to diagnose and monitor the progression in several neurological diseases. CL also represents a potential target for pharmacological strategies aimed at treating neurodegeneration. Given the equipoise that currently exists between CL metabolism, mitochondrial function, and neurological disease, we review the role of CL in nervous system physiology and monogenic and neurodegenerative disease pathophysiology, in addition to its potential application as a biomarker and pharmacological target.

## Highlights

The central nervous system is rich in lipids that support membrane fluidity, electric signal transduction, and synaptic stabilization.
Cardiolipin (CL) is a mitochondria-exclusive phospholipid essential for mitochondrial morphology, bioenergetics, dynamics, and signaling pathways.
Alterations in brain CL are associated with impaired neuronal function and neurodegeneration.
The highly tissue-specific acyl chain composition of CL confers it significant potential as a biomarker to diagnose and monitor the progression in several neurological diseases.
CL represents a potential target for pharmacological strategies aimed at treating neurodegeneration.

## Lipids and the Central Nervous System

The central nervous system (CNS) is rich in lipids, which account for approximately 50% of the total brain dry weight [1]. They are primarily localized to biological membranes, where they sustain CNS architecture and function. Sphingolipids, glycerophospholipids, and cholesterol are the predominant species and participate in a broad range of physiological functions, including cellular signaling (e.g., myelination to enable neuronal communication and nerve conduction, and lipid raft formation), energy balance, blood–brain barrier formation, and inflammatory responses [2]. It is therefore unsurprising that dysregulation of the CNS lipidome is associated with a broad spectrum of neurodegenerative disorders, such as Alzheimer’s disease (AD) and Parkinson’s disease (PD) [2].

Neuronal cellular functions have an extremely high metabolic rate, with the brain consuming up to 20% of the total body energy [2]. To fulfil this extensive energy requirement, neurons rely on glucose metabolism and mitochondrial oxidative phosphorylation (OXPHOS). Indeed, mitochondria function as sophisticated energy sensors that rapidly modulate their morphology and activity according to cellular energy demands. In addition to energy metabolism, mitochondria also participate in numerous biochemical and signaling pathways that are crucial for brain homeostasis, including cell death signaling, generation of free radical species, and lipid synthesis [3]. As a consequence of their bacterial ancestry, mitochondria have distinctive features that include multiple copies of a circular genome (mitochondrial DNA, mtDNA), the ability to divide independently from the cell, and the mitochondria-exclusive membrane phospholipid cardiolipin (CL). Despite representing just 1–3% of the total phospholipids in the CNS [1], CL is currently a major focus of neurodegenerative research.

## Cardiolipin Is a Mitochondria-Exclusive Phospholipid

**Figure 1:**
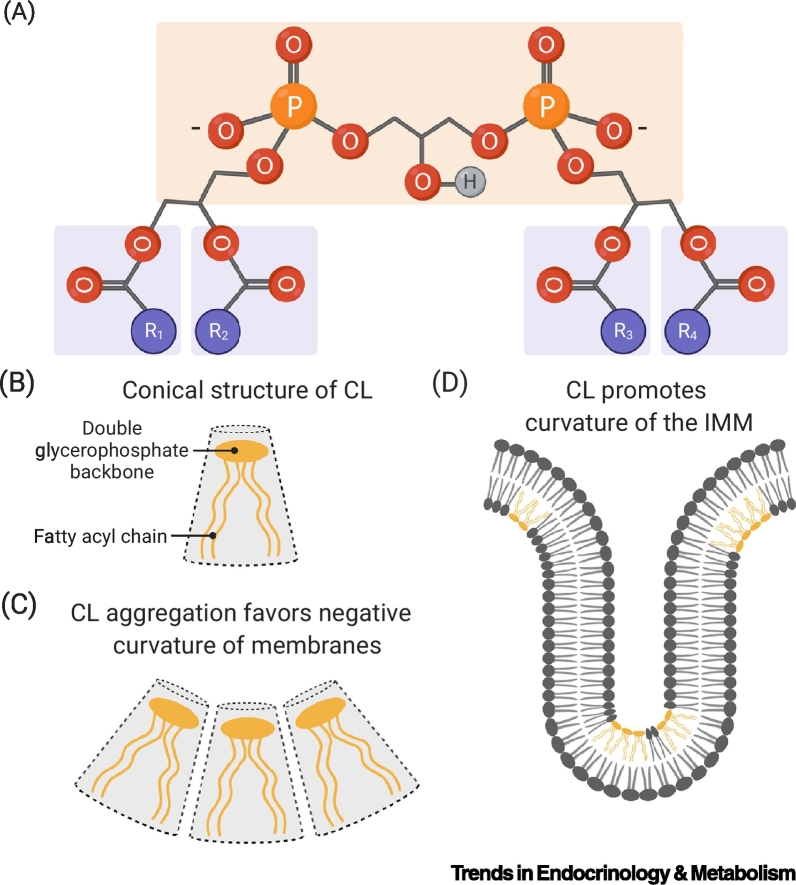
Major Characteristics of Cardiolipin (CL). (A) The chemical structure of CL is defined by its double glycerophosphate backbone and four fatty acyl chains (R_1_–R_4_). (B) The presence of a double glycerophosphate backbone and four fatty acyl side chains confers a conical shape to CL. (C) CL present in a lipid bilayer induces a negative curvature. (D) CL promotes the formation of highly curved regions within the inner mitochondrial membrane (IMM).

CL is a unique nonbilayer-forming glycerophospholipid present in the membranes of prokaryotes and the mitochondrial membranes of eukaryotes [4]. Its distinctive conical shape is defined by a double glycerophosphate backbone and four fatty acyl side chains, rather than the canonical two fatty acyl side chains commonly found in the phospholipid structure (Figure 1). Studies on human and murine tissues confirm that CL fatty acid (FA) composition is highly tissue specific [5,6]. Notably, the brain displays a unique and diverse acyl chain profile that is enriched by long-chained FA (i.e., 20:4 and 22:6), unlike other mammalian tissues (e.g., heart, skeletal muscle, and liver) that display a much more homogenous acyl chain pattern, defined by the preferential incorporation of linoleic acid (18:2) [5]. A systemic analysis of murine tissues has revealed that the diversity of CL species observed in multiple organs does not result from a stochastic process, but rather depends on the oleic (18:1) and linoleic (18:2) acid balance within individual cells. Consequently, the enrichment in longer-chained FA in the cerebrum and cerebellum might result from reduced import of FA 18:2 across the blood–brain barrier and subsequent incorporation of FA 18:1, and long-chained FA (i.e., 20:4 and 22:6) [5]. This study has opened new inroads in the tissue-specific composition of CL. However, further work is necessary to fully understand the regulatory mechanisms and biological significance of CL acyl chain diversity.

**Figure 2:**
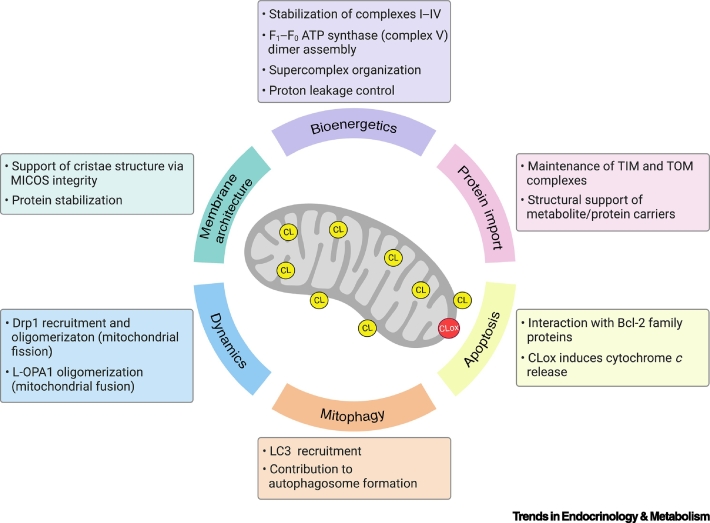
Roles of Cardiolipin (CL) in Mitochondria. CL is primarily localized within the inner mitochondrial membrane (IMM), where it contributes to maintenance of mitochondrial membrane architecture, bioenergetics, and the stability of protein carriers. In the outer mitochondrial membrane (OMM), a small fraction of CL serves as a signaling molecule for selective elimination of damaged mitochondria via mitophagy. CL is also an essential mediator of apoptosis through two mechanisms involving cytochrome *c* release that is triggered by CL peroxidation and externalization, and binding to Bcl-2 family protein Bid to induce Bax and Bak oligomerization. Finally, CL in the OMM and IMM regulates mitochondrial fission and fusion dynamics. Figure partially created with BioRender.com. Abbreviations: Bak, Bcl-2 antagonist/killer1; Bax, Bcl-2 associated X protein; Bid, BH3 interacting domain death agonist; CDP-DAG, cytidine diphosphate-diacylglycerol; CLox, oxidized cardiolipin; Drp1, dynamin-related protein 1; L-OPA1, long isoforms of optic atrophy 1; LC3, microtubule-associated protein 1A/1B-light chain 3; MICOS, mitochondrial contact site and cristae organizing system; TIM, translocase of the inner membrane; TOM, translocase of the outer membrane.

Mitochondria are characterized by a double membrane system, comprising the outer mitochondrial membrane (OMM) and the inner mitochondrial membrane (IMM). This creates two aqueous compartments, the mitochondrial intermembrane space (IMS) and a matrix region, while infolding of the IMM forms cristae [7]. The structure of mitochondrial membranes is linked to the cellular metabolic state and characterized by a unique lipid composition that sustains the energetic cellular requirements. Importantly, mitochondrial membranes contain elevated levels of phospholipids, including phosphatidylcholine (PC), phosphatidylethanolamine (PE), and CL, whereas sphingolipids and sterols are less abundant [4]. The abundance and the distribution of these phospholipids varies among mitochondrial compartments and is tightly connected to the specialized roles of different regions of the two membranes [4]. CL accounts for 10–15% of the mitochondrial phospholipid content [8] and is primarily localized in the IMM, where it is essential to maintain membrane integrity and cristae morphology [6]. In addition to its role in membrane architecture, CL participates in a wide range of mitochondrial processes, including the formation and maintenance of protein–protein and protein–membrane interactions [9,10], stabilization of the mitochondrial respiratory chain complexes (I–IV) [11., 12., 13., 14.], assembly of F_1_–F_0_ ATP synthase (complex V) dimers [15], and organization of supercomplexes [16]. Under stress, CL is externalized on the OMM and functions as a signaling molecule to promote mitophagy [17,18] and apoptotic signaling pathways [19,20]. The crucial role of CL in mitochondrial function and brain homeostasis is further highlighted by the increasing number of neurological disorders linked with CL abnormalities [21., 22., 23.] (Table 1). Moreover, the embryonic lethality of murine models deficient in enzymes involved in CL biosynthesis emphasizes the significance of CL in brain development [24,25]. A summary of the roles of CL in mitochondrial function is provided in Box 1 and illustrated in Figure 2.

**Table 1:** Cardiolipin Abnormalities in Patients with Neurological Disorders^a^

Condition	Model/biological sample	Cardiolipin abnormalities	Refs
BTHS	Skeletal muscle, heart and platelets from BTHS patients	• 80% (skeletal muscle and platelets) and 20% (heart tissue) reduction of total CL	[23]
FTD	Serum from 40 FTD patients	• ∼ 20% decrease of total CL	[22]
TBI	Brain tissue from the pericontusional area of 10 TBI patients	• Increased CLox (1 h after TBI) • Increased MLCL by hydrolysis (4 and 24 h after TBI) • Increased *TAZ* expression (4 and 24 h after TBI)	[21]

a Abbreviations: BTHS, Barth syndrome; CL, cardiolipin; CLox, oxidized CL; FTD, frontotemporal dementia; MLCL, monolysocardiolipin; TBI, traumatic brain injury; TAZ, tafazzin.

Box 1 **Roles of Cardiolipin (CL)**

**Membrane Architecture**

The unique conical shape of CL contributes towards the structural organization of mitochondria [87]. Under defined pH and ionic strength conditions, CL arranges into local hexagonal phase structures that promote the formation of highly bent regions within the IMM (see Figure 1 in the main text) [88]. In healthy cells, CL is primarily present in the IMM in close proximity to the protein complexes of oxidative phosphorylation (OXPHOS). Emerging *in vivo* and *in vitro* studies demonstrate that impaired CL biosynthesis is associated with altered cristae morphology, affecting the shape of mitochondria and their ability to adapt to cellular energy demands through OXPHOS [24,25,89,90]. The master regulator of cristae junctions and mitochondrial morphology is the mitochondrial contact site and cristae organizing system (MICOS) [91]. Recently, several proteins of the MICOS complex have been reported to interact with CL and preserve cristae architecture synergistically [92,93], thus highlighting the crucial role of CL in membrane bending and mitochondrial respiration.

**Protein Import**

Mitochondrial-related proteins have dual genomic expression; 13 OXPHOS proteins are encoded by the mitochondrial genome, while the remainder of the mitochondrial proteome (~1500 in humans) is encoded by nuclear DNA. Nuclear mitochondrial proteins are synthesized within the cytosol before they are imported to different mitochondrial compartments via multiple mechanisms [3]. The majority of such precursors gain entry into mitochondria through the translocase of the outer membrane (TOM) and translocase of the inner mitochondrial membrane 23 (TIM23) complexes. CL in the IMM and OMM (<1% of total phospholipid content [94]) is crucial to the activity and assembly of these mitochondrial translocases. Deficient CL models display perturbed TOM and TIM23 assembly, with resultant impaired protein import [10,95]. Moreover, a large body of evidence shows that CL stabilizes and preserves the activity of ADP/ATP carrier [9,96,97]. The stabilization and activity of other mitochondrial carriers, including phosphate carrier, pyruvate carrier, tricarboxylate carrier, the carnitine/acylcarnitine translocases, and calcium uniporter also require CL [98., 99., 100.]. Finally, CL is necessary for Fe-S cluster biogenesis and iron homeostasis [101], most likely through its contribution to the maturation of Yfh1/frataxin intermediate form within the mitochondrion, as observed in yeast and mammalian models of CL deficiency [102].

**Bioenergetics**

CL stabilizes the structure of mitochondrial respiratory chain complexes (I–IV) and supports efficient OXPHOS activity [11., 12., 13., 14.]. Recent cryogenic electron microscopy studies in bovine mitochondria have also revealed that CL binds tightly to the membrane domain of complex V and stabilizes its interaction with the IMM, thus controlling proton leakage and improving ATP generation, in addition to participating in dimer assembly [15]. Moreover, theoretical simulations show that transient binding of CL to complex V lubricates the ATP synthase rotor [103]. Furthermore, CL supports the organization of OXPHOS complexes into supercomplexes that, in turn, remodel and stabilize CL [104,105]. Finally, a recent study has shown that the mitochondrial ribosome binds CL, thereby stabilizing the IMM association of the protein translation machinery and supporting the biogenesis of mitochondrial OXPHOS proteins [106].

**Mitochondrial Dynamics**

Mitochondria respond to the cellular metabolic demand by modulating their number, morphology, and distribution through fragmentation (fission) and fusion events [107]. During fission, CL favors the recruitment of the GTPase dynamin-related protein 1 (Drp1) to the OMM and stimulates Drp1 activity to promote membrane remodeling and mitochondrial division [108]. Conversely, fusion of the OMM is executed by MFN1 and 2 (mitofusins), while the short (S) and long (L) isoforms of optic atrophy 1 (OPA1) perform this function for the IMM. Recently, CL has been shown to be necessary for L-OPA1 dimerization and IMM fusion [109]. Finally, CL can influence the activity of other fission-related proteins. For example, CL binds to α-synuclein, which is linked to PD, to induce mitochondrial fission and membrane fragmentation [66,68].

**Mitophagy**

In addition to mitochondrial fission and fusion, overall mitochondrial fitness is maintained through the selective elimination of damaged mitochondria by mitophagy. Mitophagy is a multistep process regulated by a consistent number of protein complexes [110]. A study conducted in neuronal cells showed that in response to mitochondrial damage, CL translocates from the IMM to the OMM to recruit microtubule-associated protein 1A/1B-light chain 3 (LC3), in addition to other proteins implicated in mitophagy, to mediate the formation of the autophagosome and fusion with lysosomes and eliminate the dysfunctional mitochondria [17,18].

Alt-text: Box 1

**Figure 3:**
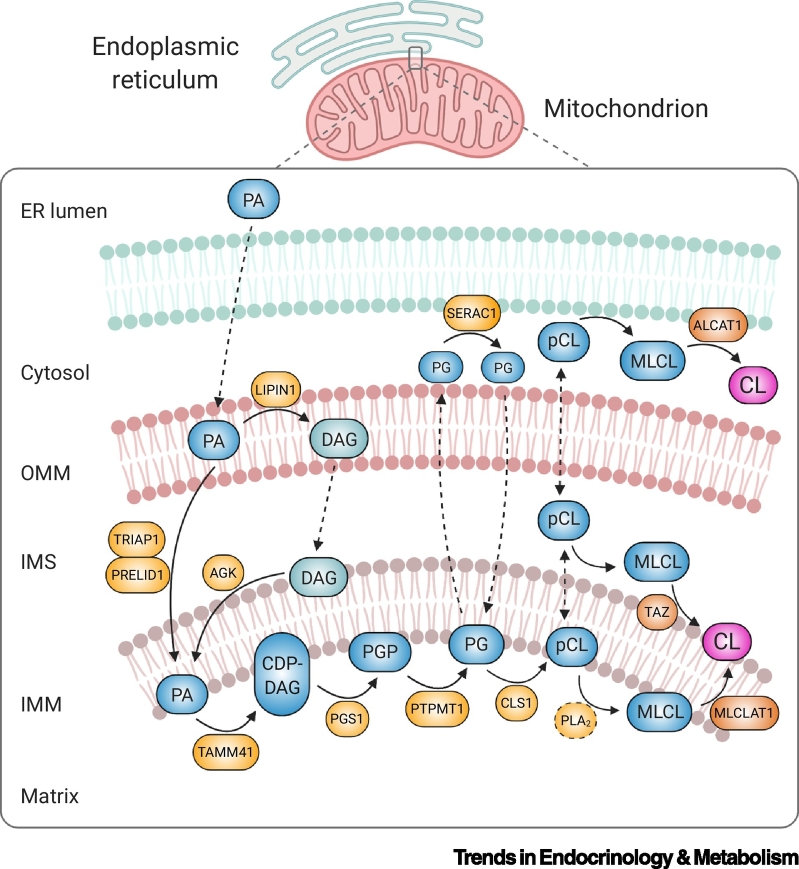
Schematic Representation of Cardiolipin (CL) Biosynthesis and Remodeling. CL is synthesized in the inner mitochondrial membrane (IMM). Biosynthesis occurs within the matrix leaflet of the IMM, while the final remodeling step can be catalyzed by three different enzymes, including tafazzin (TAZ) in the intermembrane space (IMS)-facing leaflet, monolysocardiolipin acyltransferase 1 (MLCLAT1) on the matrix-leaflet of the IMM, and acyl-CoA:lysocardiolipin acyltransferase 1 (ALCAT1) on the endoplasmic reticulum (ER). Phosphatidic acid (PA) is transported from the ER to the IMM, where it is converted to CL via a series of enzymatic reactions. PG that is generated on the matrix-leaflet of the IMM is trafficked out of the mitochondrion (broken line) and remodeled by SERAC1 on the ER side. Remodeled PG may translocate back to the matrix leaflet of the IMM (broken line) to serve as substrate for CL synthesis. PA can also be dephosphorylated into diacylglycerol (DAG) by the phosphatase LIPIN1 in the outer mitochondrial membrane (OMM). DAG is then able to traffic across the OMM (broken line) and be phosphorylated by acylglycerol kinase (AGK), forming PA in the IMS-side of the IMM. Figure created with BioRender.com. Abbreviations: CLS1, CL synthase; MLCL, monolysocardiolipin; pCL, premature CL; PG, phosphatidylglycerol; PGP, phosphatidylglycerol phosphate; PGS1, phosphatidylglycerol phosphate synthase; PLA_2_, phospholipase A_2_; PRELID1, protein of relevant evolutionary and lymphoid interest domain; PTPMT1, protein-tyrosine phosphatase mitochondrial 1; SERAC1, serine active site containing 1; TAMM41, TAM41 translocator assembly and maintenance homolog; TRIAP1, TP53-regulated inhibitor of apoptosis 1.

The endoplasmic reticulum (ER) represents the principal site of lipid biosynthesis and provides significant levels of triacylglycerol, cholesterol, phospholipids, and precursors for CL biosynthesis in the mitochondrion [26]. Thus, the communication between mitochondria and the ER via mitochondria–ER membrane contact sites is crucial to mitochondrial lipid biosynthesis and exchange [26]. In mammalian cells, *de novo* synthesis of CL (Figure 3) begins at the matrix leaflet of the IMM with phosphatidic acid (PA) [6]. PA is synthesized via several distinct enzymes that reside in the ER or on the OMM [125] and possibly in the IMM by acylglycerol kinase [126]. PA made in or transported to the OMM from the ER is moved across the IMS by the heterodimeric complex TP53-regulated inhibitor of apoptosis 1-protein of relevant evolutionary and lymphoid interest domain (TRIAP1-PRELID1) [27], before combining with cytidine triphosphate (CTP), in a reaction catalyzed by TAM41 translocator assembly and maintenance homologue (TAMM41), to generate cytidine diphosphate-diacylglycerol (CDP-DAG). CDP-DAG is fused with glycerol-phosphate by phosphatidylglycerol phosphate synthase (PGS1) to produce phosphatidylglycerol phosphate (PGP). PGP is dephosphorylated by the protein-tyrosine phosphatase mitochondrial 1 (PTPMT1) to phosphatidylglycerol (PG). Finally, PG reacts with a second molecule of CDP-DAG to form premature CL in a reaction catalyzed by CL synthase (CLS1). Mature CL is generated via the remodeling of its acyl chains, a process that is likely initiated by a phospholipase A_2_ (PLA_2_) that catalyzes the formation of the intermediate monolysocardiolipin (MLCL). MLCL is reacylated by tafazzin (TAZ) and, through a series of deacylation/reacylation reactions, the characteristic four acyl chains of mature CL are formed [6]. Two additional enzymes, monolysocardiolipin acyltransferase 1 (MLCLAT1) [28] and acyl-CoA:lysocardiolipin acyltransferase 1 (ALCAT1) [29], are also capable of attaching FAs to MLCL. Defects in genes involved in the CL biosynthetic and remodeling pathway are now a well-recognized cause of human disease. However, the components necessary for CL metabolism have not been fully resolved (indicated by broken lines in Figure 3). Thus, the true contribution of CL-related genes to human pathophysiology is unknown. A summary of the recognized CL-related monogenic disorders is provided in Box 2.

Box 2 **Barth Syndrome (BTHS) and Other Cardiolipin (CL)-Related Monogenic Disorders**

BTHS is an ultra-rare (200–300 cases worldwide) [111], X-linked recessive disorder caused by mutations in the tafazzin (*TAZ*) gene. *TAZ* encodes a transacylase that catalyzes CL remodeling within the IMM [112] (see Figure 3 in the main text). The principal clinical features of BTHS include cardiomyopathy (usually dilated, but hypertrophic cardiomyopathy has also been observed), skeletal myopathy, growth delay, and neutropenia [113]. Biochemically, an increase in MLCL:CL ratio (caused by the accumulation of MLCL and usually a decrease in CL) is universally observed in BTHS, thereby representing a reliable diagnostic biomarker for the disease [23,82,114]. Additional biochemical abnormalities observed in patients with BTHS include increased levels of plasma 3-methylglutaconic acid (3-MGC) and increased urinary 3-MGC, 3-methylglutaric acid, and 2-ethylhydracrylic acid [115].

Consistent with the multiple roles of CL in mitochondria, impaired OXPHOS, increased oxidative stress, and abnormal cristae morphology have been reported in patient-derived tissues and *in vitro* and *in vivo* models of BTHS [116]. Recently, cognitive and neurological manifestations accompanied by hippocampal degeneration have also been observed in a mouse model of BTHS, thus supporting the importance of CL in maintaining normal function of mitochondria in the CNS [117]. The complexity of BTHS, combined with the inconsistent correlation that exists between the underlying genotype and associated clinical phenotype (a variable phenotypic spectrum can exist within the same family), have contributed to the lack of approved therapies that delay the onset and/or slow progression of the disease. However, several therapeutic strategies have been investigated to alleviate cardiac dysfunction and exercise intolerance, including: (i) CL stabilization and reduction of mitochondrial dysfunction using elamipretide (Bendavia, MTP-131, SS-31), a mitochondrially targeted tetrapeptide [85]; (ii) restoration of physiological levels of mature CL via adeno-associated virus-mediated replacement of *TAZ* gene [118,119]; and (iii) stimulation of mitochondrial metabolism by increasing mitochondrial biogenesis through the repurposing of bezafibrate, an activator of peroxisome proliferator-activated receptors, which is widely used in the treatment of hyperlipidemia [120].

There are other, less well characterized, monogenic disorders linked with abnormalities in the biosynthesis and maintenance of CL and other mitochondrial phospholipids. For example, Senger syndrome is caused by mutations in an acylglycerol kinase (*AGK*) that catalyzes the synthesis of PA, an essential precursor of CL biosynthesis (see Figure 3 in the main text). However, *AGK* null cells do not exhibit CL deficiency, suggesting compensatory mechanisms exist to partially counter impaired PA production in the IMM [121]. Dilated cardiomyopathy with ataxia (DCMA, 3-methylglutaconic aciduria, type V) is an autosomal recessive disorder caused by loss of DNAJC19, an IMM protein thought to facilitate CL remodeling by TAZ, which is associated with changes in CL acyl chain composition, albeit not total CL levels [122,123]. Finally, MEGDEL syndrome (3-methylglutaconic aciduria, deafness, encephalopathy, and Leigh-like syndrome) is caused by variants in the serine active site containing 1 gene (*SERAC1*) [124], the protein product of which is implicated in the remodeling of PG, an important precursor of CL (see Figure 3 in the main text).

Alt-text: Box 2

## Cardiolipin Oxidation and Apoptosis

### Free Radical Oxidation

The brain’s high metabolic activity, regenerative limitations, and abundancy of polyunsaturated fatty acids (PUFA), which are particularly sensitive to oxidation, make it susceptible to oxidative damage. There is also extensive evidence that PUFA oxidation contributes towards the progressive neuronal loss associated with neurodegenerative diseases, such as in AD and PD [30]. The brain has therefore acquired a number of protective mechanisms against reactive oxygen species (ROS) to maintain homeostasis [31]. However, when the CNS incurs damage, the balance between ROS production and scavenging is lost, initiating a cascade of detrimental molecular events that ultimately leads to cell death and tissue damage [30]. CL is particularly susceptible to oxidative damage due to its high composition of unsaturated acyl chains, proximity to the mitochondrial electron transport chain, which is recognized as the primary source of ROS within mitochondria, and physical association with cytochrome *c*, which, in the presence of ROS, becomes a CL peroxidase [32]. Uncontrolled CL oxidation generates conformational changes that affect the physical properties of the IMM and OXPHOS activity [33]. For instance, there is evidence that oxidation of CL affects mitochondrial bioenergetics and the activity of complexes I, III, and IV [34]. Moreover, oxidized CL (CLox) favors the release of cytochrome *c* and other apoptotic factors into the cytosol, leading to cell death (see Apoptotic Signaling) [35]. Finally, the oxidized form of CL can be hydrolyzed by mitochondrial calcium-independent phospholipase A2 (iPLA2), thus triggering the production of FA second messengers, as observed in mitochondria isolated from transgenic mouse liver and cardiac tissue, which can regulate the inflammatory response [36,37].

### Apoptotic Signaling

Apoptosis is a complex mechanism of programmed cell death that is initiated by a variety of internal or external cellular events with the intention of eliminating damaged cells [38]. Mitochondria act as a critical hub for apoptotic signaling molecules because they contain cytochrome *c* and apoptosis-inducing factor (AIF), in addition to other proapoptotic factors. Recently, CL has emerged as an important player in the execution of the mitochondrial apoptotic signaling cascade via two different mechanisms. First, in the early stages of apoptosis and in the presence of ROS, CL in complex with cytochrome *c* is peroxidized in the IMM. CLox, which has a low binding affinity for cytochrome *c*, translocates from the IMM to the OMM where it initiates the apoptotic cascade most probably via the interaction with Bcl-2 family proteins, OMM permeabilization, and release of apoptotic factors into the cytosol [19]. Second, upon apoptotic stimuli, CL translocates to the cytoplasmic side of the OMM where, potentially at contact sites between the IMM and OMM, it forms a platform for caspase 8 recruitment and activation, resulting in the cleavage of the proapoptotic protein BH3 interacting domain death agonist (Bid) to its truncated form (t-Bid). This triggers oligomerization of the proteins Bcl-2 associated X protein (Bax) and Bcl-2 antagonist/killer 1 (Bak), thus inducing OMM permeabilization and cytochrome *c* release [20].

## Cardiolipin, Aging, and Neurodegeneration

Alterations in lipid metabolism have been associated with a broad range of detrimental responses in the CNS leading to neurodegeneration. Consistent with this mechanistic link, dysregulation of CL metabolism in the brain is rapidly emerging as a critical factor in the pathogenesis of several neurodegenerative states. Consequently, fully understanding the role of defective CL metabolism in nervous system homeostasis and brain function is likely to yield significant insights into the pathophysiology of neurodegenerative processes. The major CL abnormalities associated with neurodegenerative disorders are highlighted in Table 1, Table 2.

**Table 2:** Cardiolipin Abnormalities in Animal Models of Aging and Neurological Disorders^a^

Condition	Model/biological sample	Cardiolipin abnormalities	Refs
AD	Brain from 3xTg-AD mice (3 months old)	• Lower CL content in synaptic mitochondria • No change in CL saturation	[59]
Aging	Mouse brain cortex (3 and 17 months)	• 21% decrease of total CL in synaptic-mitochondria in 17-month- old mice	[41]
	Brain from 24-month-old rats	• 31% decrease of total CL	[42]
	Brain from 20–24-month-old rats	• 25% decrease of total CL	[40]
ALS	Motor cortex and spinal cord from asymptomatic (SOD1-G93A 70 days) and symptomatic (SOD1-G93A 120 days) ALS rats	• Reduced CL levels in the spinal cord of symptomatic rats	[52]
BTHS	Brain tissue from TAZ-KD mice	• Increased MLCL (19-fold) and decreased CL	[117]
PD	Brain tissue from Parkin-KO mice (2 and 24 months old)	• No change in total CL levels • CL remodeling defects with increase of short saturated CL acyl- chains in 24-month-old mice	[71]
	Brain and plasma from rats exposed to rotenone	• Increase of CLox in the substantia nigra • Increase of PUFA-containing CL in the plasma	[83]
TBI	Brain tissue and plasma from rats after controlled cortical impact (CCI)	• Decreased cortical CL (4 and 24 h after TBI) • Decreased CL in noncontusional areas (hippocampus and thalamus) • Increased in plasma levels of brain-specific CL (24 h after TBI)	[73,74,77]

a Abbreviations: AD, Alzheimer’s disease; ALS, amyotrophic lateral sclerosis; BTHS, Barth syndrome; CL, cardiolipin; CLox, oxidized CL; KD, knockdown; KO, knockout; MLCL, monolysocardiolipin; PD, Parkinson’s disease; PUFA, polyunsaturated fatty acids; SOD1, superoxide dismutase 1; TBI, traumatic brain injury; TAZ, tafazzin.

### Aging

Aging is an irreversible physiological process associated with the decline of organism cellular functions. CNS lipid metabolism has been strongly linked with aging and during senescence the brain undergoes a slow but progressive decline in lipid content [39]. Several studies have shown that murine brains display an age-dependent decrease in CL content associated with aberrant mitochondrial bioenergetics and loss of motor neurons [40., 41., 42.]. There is also evidence of increased ROS production and CL peroxidation in the brains of aged rats [40,42., 43., 44.]. However, although impaired mitochondrial function and increased ROS production represent molecular hallmarks of aging, their direct contribution remains unclear, as do the potential benefits of using antioxidants to extend lifespan [45]. Further research is therefore required to determine whether CL peroxidation has causal implications in cellular damage and aging, or whether it is part of a mitohormetic mechanism, a mitochondrial adaptive defense response triggered by exposure to mild stress, that improves mitochondrial function and longevity [46].

### Frontotemporal Dementia, Amyotrophic Lateral Sclerosis, and Overlap Syndromes

Frontotemporal dementia (FTD) is the second most common cause of early-onset dementia [47]. It is a clinically heterogenous disorder characterized by focal atrophy of frontal and temporal regions, accompanied by cognitive, behavioral, and motor impairments [48]. Several disease-causing genes associated with brain homeostasis, including chromosome 9 open reading frame 72 (*C9ORF72*), microtubule-associated protein tau (*MAPT*), progranulin (*GRN*), and fused-in-sarcoma (*FUS*), have been linked to the development of FTD and aberrant mitochondrial function [48]. Interestingly, a recent analysis of serum lipids derived from patients with FTD confirmed impaired mitochondrial bioenergetics in the setting of a significant decrease of CL content. These findings suggest a potential role for CL as a biomarker for FTD and other neurodegenerative diseases [22].

Amyotrophic lateral sclerosis (ALS) is a life-limiting neurodegenerative disorder caused by the loss of upper and lower motor neurons and consequent degeneration of the motor cortex and spinal cord [49]. The majority of ALS is sporadic. Of the 5–10% of ALS that has a genetic component, pathogenic variants are most commonly detected in superoxide dismutase 1 (*SOD1*), *C9ORF72*, *FUS*, and TAR DNA-binding protein 43 (*TARDBP*) [50]. There is increasing evidence that alterations of lipid metabolism are linked to ALS pathogenesis [51]. However, the implications of lipidome dysregulation in ALS progression remains unresolved. Using a transgenic rat model of ALS (SOD1-G93A), a recent lipidomic analysis revealed a significant decrease of CL content in the spinal cord of symptomatic ALS rats, in addition to other lipidomic changes [52]. Alterations in CL content may also mirror the loss of mitochondrial integrity observed in several ALS models [49]. Interestingly, spinal cord and, to a lesser extent, brain mitochondria of SOD1-G93A transgenic mice display increased CL peroxidation, impaired OXPHOS activity, and enhanced cytochrome *c* release from the IMM, consistent with the low binding affinity of CLox for cytochrome *c* [53]. These emerging lines of evidence suggest that aberrant CL metabolism plays a broader role in ALS pathogenesis that requires further investigation.

Clinical and molecular overlap between FTD and ALS exists with mutations in *C9orf72*, *TARDBP*, *FUS*, TANK-binding kinase 1 (*TBK1*), valosin containing protein (*VCP*), coiled-coil-helix-coiled-coil-helix domain containing 10 (*CHCHD10*), and sequestosome-1 (*SQSTM1*) [54], suggesting that the two disorders share common pathophysiological mechanisms. Interestingly, aberrant ER–mitochondria tethering has recently been observed with *TARDBP* mutations [55]. Given the central role of ER–mitochondria contact sites in lipid translocation and biosynthesis, disruption to this axis potentially has detrimental effects on lipid flux and CL biosynthesis. However, further work is required to fully understand the link between altered ER–mitochondrial signaling, CL content/metabolism, and FTD/ALS pathophysiology.

### Alzheimer’s Disease

AD is the most prevalent cause of dementia in aging people [56]. Clinically, the disease is characterized by a progressive loss of memory, decline of learning abilities, disorientation, and mood swings [56]. Over the past three decades, substantial evidence has been generated to support the notion that the synaptic loss and neuronal impairment observed in AD is causally related to the co-presence of extracellular amyloid-ß plaques and toxic neurofibrillary tangles of tau protein in the brain [56]. Moreover, the abnormal accumulation of misfolded tau protein and amyloid-ß aggregates observed in familial AD (<5% of cases) have been associated with mutations in the amyloid precursor protein (*APP*) and presenilin 1 and 2 (*PSEN1* and *2*) [57]. By contrast, sporadic AD, which occurs with much higher prevalence, is hypothesized to result from complex interactions between genetic, environmental, and lifestyle factors [56]. Nevertheless, the complexity of AD suggests that additional players are involved in the pathogenesis of the disease. Changes in the brain and plasma lipidome have been extensively observed in people with AD [58]. However, knowledge concerning the role of CL is limited. A reduction in total CL, associated with mitochondrial synaptic dysfunction and oxidative stress, has been observed in a mouse model of AD, suggesting a contribution of CL to the disease pathogenesis [59]. Additionally, *in vivo* and *in vitro* models of AD have shown that mitochondrial membranes are particularly vulnerable to tau aggregates, leading to neuronal toxicity [60], and that tau protein preferentially binds to CL-rich regions of the OMM, inducing mitochondrial swelling, cytochrome *c* release, and decreased membrane potential [61]. Finally, a critical driver of AD is the aberrant neuroinflammatory response, induced by chronic microglial activation and release of proinflammatory cytokines, and a potential protective role of CL in the AD inflammatory response has been proposed [62]. Indeed, CL extracellularly released by damaged cells may regulate microglia function by upregulating the phagocytosis of amyloid-ß deposits, thus enhancing neuronal survival [62]. Collectively, these observations suggest a causal association between CL and AD pathophysiology that requires further study.

### Parkinson’s Disease

PD is characterized by the progressive loss of dopaminergic neurons in the substantia nigra and the presence of intraneuronal aggregates of misfolded α-synuclein. The majority of PD is idiopathic, with familial PD accounting for 5–10% of cases [63]. Several molecular factors contribute to the pathogenesis of PD, including toxic α-synuclein deposition, oxidative stress, and impaired mitochondrial function (i.e., biogenesis, bioenergetics, and mitophagy) [63]. Numerous *in vitro* and *in vivo* studies confirm that the neurotoxicity of the intracellular aggregates is associated with the ability of misfolded α-synuclein to interact with CL, thus impacting mitochondrial membrane integrity [64,65]. Moreover, the co-presence of CL and α-synuclein oligomers favors the formation of ion-permeable pores, inducing mitochondrial membrane permeabilization and cytochrome *c* release [66], in addition to disrupting bioenergetics [64,67]. However, CL exposure in the OMM can also promote α-synuclein refolding, thereby reducing the toxicity of the intracellular aggregates, preventing neuronal loss [68]. Indeed, α-synuclein can form a triple complex with CL and cytochrome *c*, which prevents cytochrome *c* release and delays neuronal damage [69]. Reduced α-synuclein oligomerization may also be facilitated by the inhibition of ALCAT1, an enzyme involved in pathologic CL remodeling and formation of highly oxidizable CL species [70] (Figure 3). Abnormalities in CL content have also been associated with impaired mitophagy [18,71] and defects of complex I activity [72] in multiple PD models. Together, these data confirm an important role for CL in PD. However, further work is necessary to determine the precise balance between the detrimental and protective implications of CL in PD pathogenesis.

### Traumatic Brain Injury

Traumatic brain injury (TBI) is the leading cause of death in young adults. From a pathophysiological perspective, the neurological damage that occurs is divided into primary and secondary injuries. Primary brain injuries derive from the immediate mechanical impact, whereas secondary injuries occur within hours or weeks from the initial insult as a consequence of a wide range of signaling responses, including oxidative stress, lipid peroxidation, apoptosis, and inflammation [35]. In a controlled cortical impact (CCI) rat model of TBI, loss of CL content has been observed post-TBI in the contusional and pericontusional regions [73], while the unique long-chain PUFA of CL present in the brain are readily oxidized (CLox) [74., 75., 76.], hydrolyzed, and released into the systemic circulation, where they activate a large number of well-characterized inflammatory signaling molecules [21]. Consequently, these oxidized FAs have been proposed as mediators and/or regulators of the inflammatory response to the injury [74]. Moreover, given that in murine models brain CL is detectable in the blood within hours of the insult, it potentially represents a biomarker to assess the severity and progression of the injury [77]. The procoagulant effects of CL present in circulating brain-derived microparticles have also been linked with TBI-associated coagulopathy in mice, a feature of TBI that is associated with a poorer outcome in humans [78]. Finally, preliminary evidence suggests that CL is externalized from the IMM to the OMM following TBI, thereby eliminating damaged brain mitochondria through mitophagy. This early protective mechanism is activated during the first few hours after the injury before rapidly being replaced by uncontrolled apoptotic signaling pathways. Accordingly, the development of small molecules that modulate the mitophagy response might prevent the extensive neuronal loss that occurs, thereby representing a potential therapeutic strategy in TBI [79].

## Management and Treatment Strategies

Dysregulated phospholipid levels in the brain and serum have been associated with acute and chronic pathological conditions. The development of liquid chromatography-tandem mass spectrometry (LC-MS/MS) lipidomics approaches have already generated important data concerning altered CL species in human and animal models of neurodegenerative diseases [59,80,81]. Detection of reduced CL content and increased MLCL expressed as a ratio of MLCL:CL is currently used in clinical practice as a diagnostic biomarker for Barth syndrome (BTHS) [82]. Furthermore, increasing information in humans and in animal models indicates that CL species detected in the systemic circulation have the potential to act as biomarkers that measure disease progression in other neurological disorders, for example, FTD, PD, and TBI [22,74,83]. A summary of the CL species detected in the serum of human and animal models is summarized in Table 1, Table 2, respectively.

Rapid advances in current understanding of the novel roles of CL in brain homeostasis and neurodegeneration represent an exciting opportunity to develop new treatment strategies aimed at targeting various neurodegenerative processes. Small molecules that preserve mitochondrial CL content and integrity have the potential to prevent and/or attenuate the mitochondrial damage associated with neurodegeneration. For example, elamipretide (Bendavia, MTP-131, SS-31) is a mitochondrial-targeted synthetic tetrapeptide that concentrates at the IMM and binds to 12 CL-binding proteins [84]. While the precise molecular mechanism is unclear, elamipretide is proposed to stabilize CL, thus improving OXPHOS efficiency. A Phase III trial of elamipretide in primary mitochondrial myopathy did not meet its primary end-points. However, statistically significant improvements in exercise tolerance, measured using a 6-minute walk test, and BTHS symptom scores at 36 weeks were recorded during the open label extension of a Phase II/III study in BTHS [85]. There is also promising preclinical data for elamipretide as a disease-modifying molecule in TBI [86].

## Concluding Remarks

Over recent years, clinical and experimental studies from human and animal models have provided compelling evidence that links aberrant CL metabolism with neurological dysfunction. Alterations in CL profiles measured in several neurodegenerative disorders further emphasizes the important role of CL in brain homeostasis and neuronal physiology. Given the equipoise that currently exists between CL, mitochondria, and neurological disease, there is critical need to fully understand the factors that regulate CL acyl chain composition and whether abnormalities in CL are a direct cause, or downstream effect, of the underlying pathological process. Moreover, despite significant advances in lipidomic techniques and *in vivo* approaches, the pathways involved with normal CL metabolism remain poorly understood (see Outstanding Questions). Consequently, CL may still contribute to numerous, as yet uncharacterized, aspects of cellular homeostasis and human health. In future, the application of innovative, multi-research domain approaches will be crucial when evaluating these roles and their contribution to nervous system homeostasis and brain function.

Outstanding Questions What is the functional role of cardiolipin (CL) long-chained fatty acids in the brain?

What are the missing steps in the CL biosynthetic pathway?

What are the molecular mechanisms underpinning aberrant CL composition in human disease states?

Could CL represent a biomarker and pharmacological target in CL-related monogenic and neurodegenerative disorders?

Alt-text: Outstanding Questions
